# Resistance training and cardiovascular health: epigenetic regulation

**DOI:** 10.3389/fphys.2025.1701689

**Published:** 2026-01-15

**Authors:** João Gabriel Silva, Luis Felipe Rodrigues, Thyerre Torres, Alex Cleber Improta-Caria, Edilamar Menezes Oliveira, Tiago Fernandes

**Affiliations:** 1 Laboratory of Biochemistry and Molecular Biology of the Exercise, University of Sao Paulo (USP), SãoPaulo, Brazil; 2 University Center for Science and Entrepreneurship, Santo Antoniode Jesus, Brazil

**Keywords:** cardiac remodeling, cardiovascular health, DNA methylation, epigenetic regulation, gene expression, histone modification, non-coding RNAs, resistance training

## Abstract

Resistance training plays a crucial role in cardiovascular health by promoting epigenetic adaptations that beneficially modulate gene expression. These modifications include DNA methylation, histone alterations, and regulation by non-coding RNAs, which directly affect cardiac muscle and the vascular system. Such epigenetic changes lead to improved cardiac function, reduced inflammation, optimized metabolism, and protection against cardiovascular diseases. Resistance training induces the release of signaling molecules that mediate favorable systemic adaptations. Studies demonstrate that resistance training, especially when combined with aerobic training, improves cardiovascular risk factors such as blood pressure and lipid profile. Epigenetic regulation is fundamental to the plasticity of the cardiovascular system and the reversibility of exercise-induced adaptations. Although extreme exercise may pose risks, regular and moderate resistance training is safe and effective in the prevention and management of cardiovascular diseases through these molecular mechanisms. Further investigation into these epigenetic modifications may unveil novel exercise-based therapeutic strategies to enhance cardiovascular health.

## 1 Introduction

Resistance training (RT) is widely recognized for improving health, physical fitness, and athletic performance, typically involving the use of free weights, body weight, or specialized equipment (Westcott, 2012). Numerous studies have demonstrated its benefits in increasing strength and muscle mass, enhancing quality of life, and preventing various diseases (Prestes et al., 2019; Peng et al., 2024; Vann et al., 2020). For instance (Shailendra et al., 2022), reported an association between RT and reduced risk of all-cause, cardiovascular, and cancer-specific mortality, as well as improved health-related quality of life in individuals with rheumatic diseases. Beyond its musculoskeletal effects, RT also contributes to the functional improvement of organs such as the heart, vasculature, and lungs (Paluch et al., 2024; De Caterina et al., 2019).

In the cardiovascular system specifically, reviews indicate that RT enhances hemodynamic and contractile function and improves overall cardiac metabolism, both in healthy individuals and in those with cardiovascular disease (Liu et al., 2019; Tucker et al., 2022). This is particularly relevant given that cardiovascular diseases remain the leading cause of death worldwide, and interest in non-pharmacological therapeutic strategies continues to grow (Pillon et al., 2020). At the molecular level, exercise—especially aerobic training—is known to induce beneficial changes in gene and protein expression (Popov et al., 2018; Miranda Furtado et al., 2024); however, relatively few studies have examined the effects of RT on epigenetic mechanisms such as DNA methylation, histone modifications, and non-coding RNAs (ncRNAs) (Plaza-Diaz et al., 2022; Weineck, 1999).

Despite the recognized importance of RT, molecular and epigenetic investigations of its adaptive cardiovascular effects remain limited, highlighting the need for further research (Powers, 1999). Therefore, the aim of this review is to explore the effects of RT on epigenetic regulatory mechanisms within the cardiovascular system in both humans and animal models.

## 2 Impact of RT on the cardiovascular system

Over the years, several benefits generated by RT have been demonstrated (Zouita et al., 2023; Lacio et al., 2021), particularly in skeletal muscle, where it promotes hypertrophy and maintenance of strength levels (Alcaraz-Ibañez and Rodríguez-Pérez, 2018; Bachero-Mena et al., 2019). These adaptations are crucial not only for athletic and functional performance (Berin et al., 2022; Krzysztofik et al., 2019; Gylling et al., 2020), but also for maintaining quality of life in healthy individuals (Abou Sawan et al., 2023; Hunter et al., 2004). Importantly, in the context of aging, adequate muscle strength and mass are strongly associated with greater autonomy, reduced risk of falls, prevention of systemic and mental diseases, and increased life expectancy (de Sousa Ferreira et al., 2022; Cardo et al., 2024).

Beyond muscular adaptations, RT induces significant metabolic improvements. Aristizabal et al. (Aristizabal et al., 2015) demonstrated an increased basal metabolic rate after 9 months of training using dual-energy X-ray absorptiometry (DEXA). RT also enhances insulin sensitivity and glycemic control (Bronczek et al., 2021), leading to improved intramuscular glycogen storage (Correia et al., 2023). These findings indicate that RT acts as a metabolic modulator, supporting both energy efficiency and glucose homeostasis.

Clinically, RT contributes to the prevention and management of chronic diseases such as hypertension and diabetes mellitus (Hearris et al., 2018). In diabetic patients, RT acutely reduces serum glucose levels and chronically enhances GLUT4 expression and insulin sensitivity—key mechanisms for glucose metabolism and glycemic stability (Correia et al., 2023; Lima et al., 2022). Regarding hypertension, Moraes et al. (Flores-Opazo et al., 2020) reported that just 12 weeks of RT significantly reduced blood pressure in grade 1 hypertensive patients. Complementary evidence shows that performing RT 2–3 times per week with moderate to high loads can sustain these reductions for up to 14 weeks after cessation of training (Moraes et al., 2012; Ferreira Mendes et al., 2024), underscoring its long-term cardiovascular benefit.

Another crucial adaptation involves the cardiovascular system itself. RT induces physiological cardiac remodeling, often described as concentric cardiac hypertrophy, initially conceptualized by Morganroth et al. (1975), Correia et al. (2023), Mihl et al. (2008). In contrast to the eccentric hypertrophy associated with aerobic exercise—characterized by increased chamber volume and sarcomeres in series—RT produces concentric hypertrophy, defined by increased wall thickness and sarcomeres in parallel (Mihl et al., 2008; Morganroth et al., 1975). Although Mongaroth’s model provided an early framework, subsequent research has revealed a more complex and integrated picture of cardiac remodeling (Haykowsky et al., 2018; Fernandes et al., 2011), including the contribution of the right ventricle and transmural pressure gradients often neglected in early studies.

Recent investigations reinforce that RT, when properly prescribed, promotes beneficial cardiovascular remodeling without pathological hypertrophy. Pamart et al. (2025), Fernandes et al. (2011) reported reductions in blood pressure and heart rate after 20 weeks of high-intensity RT, alongside moderate biventricular Pamart remodeling and preserved cardiac function. In a murine model, Li et al. (2021), Haykowsky et al. (2002) demonstrated that RT improved vascular function and enhanced mitochondrial biogenesis after myocardial infarction, suggesting a direct cardioprotective effect through improved cardiac metabolism.

At the vascular level, RT stimulates angiogenic processes that contribute to improved perfusion and vascular health. Both animal and human studies have shown that RT protocols induce angiogenic responses and can aid in the management of vascular diseases (Naylor et al., 2008; Pamart et al., 2025). Traditional RT and blood flow restriction training (Kaatsu-training) have been shown to activate transcription factors and genes related to angiogenesis (Li et al., 2021; McIntosh et al., 2024), increasing vascular density in healthy and clinical populations. This occurs through elevated expression of nitric oxide and vascular endothelial growth factor (VEGF), leading to localized vascular expansion. Although the restriction method is limited to limbs and often associated with local discomfort, it allows the use of low loads (low % of 1RM), making it suitable for populations unable to tolerate high mechanical stress, such as elderly or obese individuals (Banks et al., 2024).

**FIGURE 1:**
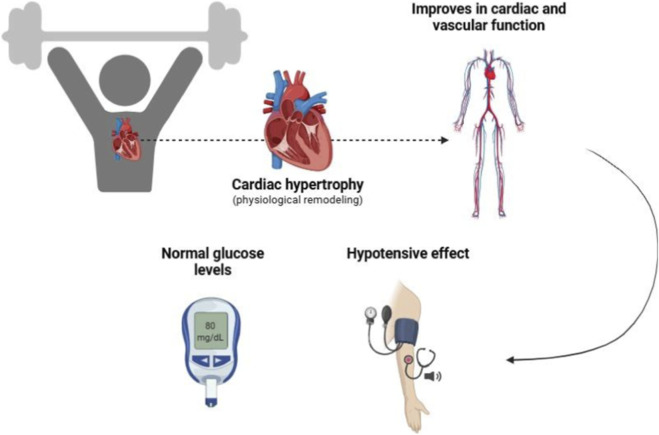
Benefits of RT on cardiovascular system. Regular RT can improve cardiovascular efficiency, boost its pumping ability, and result in physiological cardiac hypertrophy, along with lower blood pressure, improved lipid profiles, and enhanced insulin sensitivity.

Taking together, these findings demonstrate that RT exerts systemic effects extending beyond skeletal muscle, with direct implications for cardiovascular health, as shown in Figure 1. While acute RT sessions may transiently elevate blood pressure and cardiac workload, chronic adaptations include reduced resting blood pressure, improved endothelial function, and enhanced autonomic regulation. When implemented with appropriate progression and supervision, RT becomes a safe and effective therapeutic strategy for cardiovascular disease management (Yang K. et al., 2024). Furthermore, improved nitric oxide bioavailability, decreased endothelin-1, and reduced oxidative stress contribute to enhanced vasodilation, improved hemodynamic control, and reduced arterial stiffness (Yang J. et al., 2024).

## 3 Impact of RT on the signaling pathways

In recent years, considerable efforts have been directed toward a better understanding of the cellular and molecular signaling pathways underlying the adaptations induced by RT, particularly those regulating muscle hypertrophy and protein degradation control (Perera et al., 2022). This knowledge is fundamental not only for understanding physiological responses in healthy individuals but also for elucidating mechanisms involved in skeletal muscle disorders such as fibromyalgia and sarcopenia (De Matos et al., 2024; Sartori et al., 2021). Expanding comprehension of the regulatory mechanisms that orchestrate these signaling pathways is therefore essential to explain the molecular determinants of phenotypic adaptations observed in response to RT (Schiaffino et al., 2021). In addition to classical intracellular cascades, increasing evidence highlights the importance of epigenetic regulation—including DNA methylation, histone acetylation/deacetylation, chromatin remodeling, and non-coding RNAs—in modulating the transcriptional programs activated by RT. Notably, RT-induced alterations in histone deacetylase (HDAC) activity, the AMPK–SIRT axis, and DNA methyltransferase–dependent methylation patterns have emerged as key mechanisms influencing gene expression, cellular growth, and metabolic remodeling.

In this context, Wang et al. (Pascual-Fernández et al., 2020) demonstrated through skeletal muscle biopsy that RT can activate signaling pathways associated with mitochondrial biogenesis, suggesting a possible interaction between mitochondrial regulatory pathways and those mediated by mTOR. As the understanding of muscle hypertrophy has evolved, evidence now indicates that increases in muscle cross-sectional area occur through three main mechanisms: the formation of syncytial fibers, the addition of myonuclei, and the expansion of cytoplasmic volume, characterized by elevated water content, energy substrates, and fibrillar organelles (Attwaters and Hughes, 2022). Complementarily, epigenetic processes such as HDAC inhibition particularly of class IIa isoforms facilitate MEF2-mediated transcriptional activation, while the AMPK–SIRT1 axis modulates mitochondrial and metabolic gene networks through NAD^+^-dependent deacetylation. RT may also influence DNMT-mediated methylation of promoters related to inflammatory control, structural remodeling, and insulin sensitivity, reinforcing its capacity to induce long-term phenotypic adaptations (Fakhar et al., 2025).

Although most mechanistic insights come from skeletal muscle, the cardiovascular system has also been investigated to a lesser extent. Current evidence suggests that the pressure-induced hemodynamic overload generated during RT resembles, in part, the mechanical stress experienced in early stages of certain cardiovascular diseases, such as hypertension. Both conditions may share transient molecular responses, including increased fibrotic signaling and extracellular matrix remodeling, before pathological decompensation occurs (Colorado et al., 2023). Importantly, the overload imposed during RT is intermittent and physiological, differing fundamentally from the chronic and pathological nature of hemodynamic stress in disease states (Wang et al., 2011). This distinction is crucial, as it explains the reversibility and adaptive potential of exercise-induced remodeling (Bachero-Mena et al., 2019; Furrer and Handschin, 2024).

Advances in next-generation sequencing have substantially expanded knowledge of the genomic and molecular interactions involved in RT-induced adaptations, enabling a more integrative view of the signaling networks that support the cardioprotective and systemic effects of physical exercise (Melo et al., 2018; Martinez et al., 2021; Rodríguez et al., 2024). Among these, the PI3K/AKT/mTOR pathway stands out as one of the most well-characterized cascades activated by RT. This pathway is pivotal for protein synthesis, cellular growth, proliferation, and survival (Bouchard, 2019). In cardiac tissue, PI3K/AKT signaling has been closely associated with physiological remodeling induced by exercise; however, most studies to date have focused on aerobic training models (Smith, 2021).

Notably, RT preferentially modulates signaling pathways related to tissue morphology and extracellular matrix remodeling (Oliveira-Junior et al., 2022), whereas aerobic exercise predominantly alters metabolic profiles associated with cardiac function (Fukada and Ito, 2021). Beyond these classic pathways, RT also influences systemic metabolic signaling, particularly through cytokines such as interleukin-6 (IL-6), which acts as a key mediator in the hypertrophic response and in the regulation of inflammation (Soci et al., 2016). Epigenetic regulators, including non-coding RNAs, play an additional role in modulating these processes by influencing mRNA stability and translation of transcripts related to hypertrophy, fibrosis, and angiogenesis (da Costa et al., 2015).

In summary, RT orchestrates a complex network of molecular signaling pathways that extend beyond skeletal muscle to other tissues, including neural, immune, and adipose systems. Integrated with epigenetic mechanisms that fine-tune gene accessibility and transcription, these interactions contribute to the systemic adaptations and broad health benefits associated with RT.

## 4 Impact of RT on the epigenetic regulations

When we talk about regulatory mechanisms of signaling pathways, epigenetics is a field of knowledge that covers this topic by providing mechanisms and changes in gene expression without altering the biological sequence of DNA (Fukada and Ito, 2021). Currently, the literature identifies three major epigenetic mechanisms: DNA methylation, histone modification, and non-coding RNAs (da Costa et al., 2015; Raleigh, 2021; Wu et al., 2023); the latter being considered the main class of gene regulators already described, representing nearly 80% of the human genome (Wu et al., 2023). Importantly, RT induces both acute epigenetic responses, which occur within minutes to hours after a single exercise session, and chronic epigenetic adaptations, which accumulate over repeated training bouts and contribute to long-term phenotypic remodeling. Acute responses are typically characterized by transient changes in gene expression, rapid chromatin accessibility shifts, and short-term fluctuations in DNA methylation or histone acetylation status. In contrast, chronic adaptations are associated with more stable methylation patterns, persistent histone signatures, and long-lasting miRNA expression profiles that collectively contribute to the establishment of exercise-induced molecular memory (Wu et al., 2023).

The literature has shown that a single session or a RT program can alter DNA methylation sites, which is closely linked to the regulation of gene expression, given that unmethylated DNA regions (euchromatin) allow full action of RNA polymerase II (Nurk et al., 2022; Bagley et al., 2020; Bittel and Chen, 2024). In this context, the premise of investigating the relationship between hyper- or hypomethylated regions and their relationships with gene expression—especially in skeletal muscle—was highlighted by Turner et al. (2019), Lindholm et al. (2014), who introduced the concept of an epigenetic memory, demonstrating that exercise training acts as a chronic modulator of gene expression in trained individuals compared to sedentary controls. In an extensive review, Bittel et al. (2024) reinforced the role of DNA methylation in the adaptive response to physical exercise, emphasizing the importance of methylation in promoter, gene body, and enhancer regions in the regulation of skeletal muscle gene expression, while also highlighting individual factors (e.g., genetics and diet) in shaping the epigenetic response (Petrie et al., 2020). Recent studies have identified specific methylation changes in genes such as PPARGC1A, NR4A3, and TFAM following both resistance and endurance exercise, associated with enhanced mitochondrial biogenesis and oxidative metabolism (Petrie et al., 2020). Moreover, AMPK–PGC-1α and MAPK signaling pathways appear to mediate many of these methylation-dependent transcriptional effects. In contrast, cardiac-specific evidence remains limited, but emerging data suggest that aerobic exercise promotes demethylation of genes involved in fatty acid oxidation (PPARα, CPT1B) and calcium handling (SERCA2a), contributing to improved metabolic and contractile function (Geiger et al., 2024).

**FIGURE 2:**
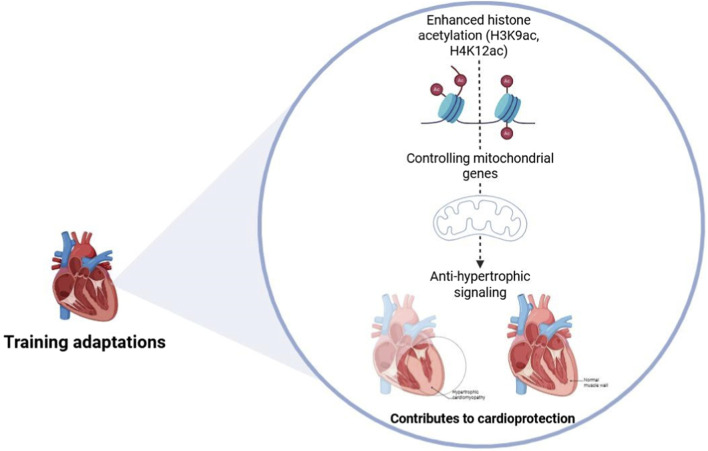
Epigenetic regulations in exercise training-induced cardioprotection. The figure illustrates how enhanced histone acetylation (H3K9ac, H4K1the) regulates mitochondrial genes and promotes anti-hypertrophic signaling. These changes contribute to cardioprotection, distinguishing between pathological and physiological cardiac conditions.

Histone modification has also been a significant area of investigation in recent years. Physical exercise has been shown to modulate histone acetylation and deacetylation, processes integral to the regulation of gene expression and consequently to adaptations induced by training (Bittel and Chen, 2024; Turner et al., 2019). Acute responses often include rapid histone acetylation changes at promoters of metabolic and stress-responsive genes, whereas chronic training appears to consolidate these modifications into more stable chromatin states that support long-term transcriptional reprogramming. Recent reviews indicate that histone modifications (acetylation, methylation, phosphorylation) play a crucial role in muscular adaptations to exercise, including RT, by regulating transcription of genes involved in hypertrophy and metabolism (Wu et al., 2023; McGee et al., 2009). In skeletal muscle, increased H3K4me3 and decreased H3K27me3 have been observed at loci governing energy metabolism and muscle remodeling (Lim et al., 2020). In cardiac tissue, aerobic exercise has been linked to enhanced histone acetylation (H3K9ac, H4K12ac) in regions controlling mitochondrial genes and anti-hypertrophic signaling, suggesting that exercise-driven chromatin remodeling contributes to cardioprotection (Wu et al., 2021), as shown in Figure 2. These findings reinforce the need to distinguish between skeletal and cardiac muscle evidence, as many exercise–epigenetics studies rely on skeletal muscle samples and cannot be directly extrapolated to the heart.

Non-coding RNAs were discovered through important discoveries in the 1970s and 1980s, when studies on genomic complexity revealed no correlation between genome size and organismal complexity—a phenomenon known as the C-value paradox (Wu et al., 2021; Seaborne et al., 2018). This paradox led to the recognition that large portions of the genome could perform regulatory functions beyond the classical “DNA–RNA–protein” framework proposed by Francis Crick in 1958. Initially regarded as “junk DNA,” these regions were later shown to exert key regulatory and catalytic roles as new molecular tools enabled their functional characterization (Finke et al., 2022).

Currently, ncRNAs are considered one of the largest classes of gene regulators. Even without coding for proteins, they regulate pre- and post-transcriptional control, chromatin remodeling, histone modification, and cell cycle regulation (George et al., 2024; Jarroux et al., 2017; Li, 2023; Yin and Shen, 2023; Li et al., 2024). They are generally divided into short and long non-coding RNAs (lncRNAs), with the former including miRNAs, the best-characterized subgroup (Loganathan and Doss, 2023; Shang et al., 2023; He et al., 2023). MiRNAs are small non-coding RNAs widely studied for their post-transcriptional regulatory roles (Zhang et al., 2019; O’Brien et al., 2018; Improta-Caria et al., 2024). Acute RT sessions can produce transient fluctuations in circulating and intramuscular miRNA levels, whereas chronic RT leads to more stable remodeling of miRNA networks that regulate long-term hypertrophic and metabolic pathways. However, limited data exist on the role of miRNAs in cardiac muscle in response to RT. Some miRNAs exhibit tissue-dependent expression and can be released into systemic circulation in response to exercise, influencing pathways related to muscle function and cardiovascular health (Romaine et al., 2015; Fóthi et al., 2020; Ma et al., 2025; Liu et al., 2017; Wang et al., 2018). Certain miRNAs regulate adaptations in skeletal muscle and myocardial crosstalk during exercise, thus promoting systemic cardiovascular benefits (Karunakaran and Rayner, 2013; dos Santos et al., 2022).

Updates from the American Heart Association emphasize the importance of RT for individuals with and without cardiovascular disease (CVD). RT not only enhances muscle strength but also improves cardiovascular health by optimizing hemodynamics and reducing CVD risk factors. Combining aerobic and RT modalities may yield synergistic cardiovascular benefits (Cui et al., 2017; Nappi et al., 2023).

**FIGURE 3:**
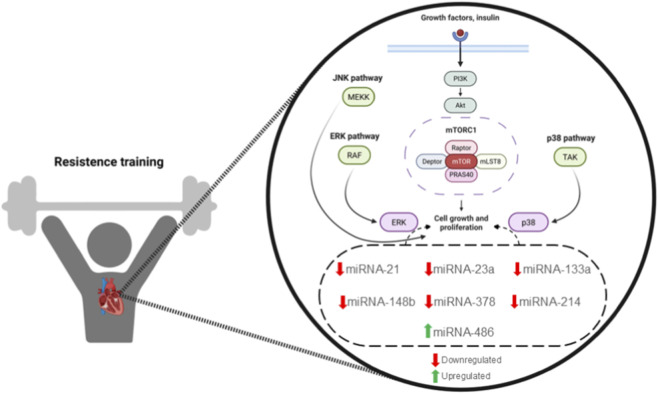
Epigenetic regulation is induced by RT. This figure illustrates the relationship between RT and various signaling pathways (JNK, ERK, p38) that are influenced by growth factors and insulin. It highlights the regulation of specific miRNAs (miR-21, miR-23a, miR-133a, miR-148b, miR-214, and miR-486), showing which are upregulated or downregulated in response to RT. These changes play a significant role in cellular growth and proliferation, contributing to muscle adaptation and cardiovascular health.

MiRNAs regulate key signaling pathways, including the IGF1/PI3K/AKT/mTOR axis, which is essential for exercise adaptation (Zhou et al., 2018; Zhang et al., 2017). Following exercise, miRNAs such as *miR-23a*, *miR-133a*, and *miR-378* show marked expression changes in muscle tissue. *miR-23a*, for instance, is downregulated 2–4 h post-exercise, suggesting a role in muscle protein metabolism (Domańska-Senderowska et al., 2019). *miR-486* has been associated with mitochondrial adaptation, while *miR-21* and *miR-148b* regulate insulin signaling during RT (Zhou et al., 2020). RT also decreases *miR-214* expression, which leads to increased *SERCA2a* expression and improved cardiomyocyte contraction and relaxation. This mechanism is associated with physiological cardiac hypertrophy, demonstrating that RT influences the cardiac phenotype through miRNA-mediated regulation (Zhou et al., 2020), as shown in Figure 3.

Importantly, emerging evidence suggests that these epigenetic modifications extend beyond skeletal muscle and may contribute to cardiovascular adaptations commonly observed with exercise. Alterations in DNA methylation and histone acetylation status in vascular and cardiac tissues have been associated with improved endothelial function through enhanced expression of nitric oxide–related genes and reductions in pro-inflammatory signaling, processes that collectively support better vasodilation and lower arterial stiffness. Likewise, miRNA-mediated regulation of calcium-handling proteins, mitochondrial genes, and anti-fibrotic pathways—such as changes in miR-133a, miR-486, and miR-214—has been linked to improved cardiomyocyte contractility, physiological hypertrophy, and enhanced metabolic efficiency, which together contribute to increases in VO_2_peak. Although direct evidence in response specifically to RT remains limited, the available literature indicates that epigenetic modulation represents a mechanistic bridge connecting exercise stimuli to favorable cardiovascular remodeling and function (Domańska-Senderowska et al., 2019).

In addition to the epigenetic mechanisms modulated by RT, individual factors such as age, sex, metabolic status, obesity, and pre-existing cardiovascular disease play a decisive role in shaping the magnitude and direction of these responses. Older adults, for instance, typically exhibit higher global DNA methylation and altered HDAC and sirtuin activity, which may reduce epigenetic plasticity and influence exercise-induced adaptations. Sex-related differences also affect the expression of key miRNAs and hormone-driven pathways that interact with SIRT1, AMPK, and DNMTs, potentially resulting in distinct cardiovascular outcomes between men and women. Likewise, conditions such as insulin resistance, low-grade inflammation, and excess adiposity modify the cellular metabolic environment, impacting bioenergetic status and, consequently, the efficiency of epigenetic mechanisms dependent on redox balance and NAD^+^/NADH availability. In individuals with established cardiovascular disease, pre-existing epigenetic remodeling may either attenuate or enhance responsiveness to training stimuli. Considering these variables is essential to understanding the heterogeneity of molecular adaptations and strengthening the translational relevance of exercise-induced epigenetic effects (Zhou et al., 2020; Domańska-Senderowska et al., 2019).

Another class of non-coding RNAs, the lncRNAs, still requires deeper exploration regarding their roles in RT. However, they have been shown to regulate skeletal muscle metabolism. Melo et al. (2015) identified a muscle-specific lncRNA, *linc-MD1*, which modulates the timing of muscle differentiation by acting as a competing endogenous RNA (ceRNA) in mouse and human myocytes. *Linc-MD1* binds to *miR-133* and *miR-135*, thus regulating transcription factors such as *MAML1* and *MEF2C*, which activate muscle-specific genes. Modulating *linc-MD1* levels can delay or accelerate muscle differentiation, demonstrating its essential role in muscle plasticity (Cesana et al., 2011). However, studies on the effects of RT on lncRNAs expression in cardiac tissue still need to be addressed.

Critical considerations regarding the current literature are also necessary to contextualize the epigenetic adaptations induced by RT. Although several studies report consistent changes in DNA methylation, histone modifications, and non-coding RNA expression, conflicting results remain, particularly regarding the magnitude, direction, and functional significance of these epigenetic shifts. These discrepancies arise partly from methodological limitations, including small sample sizes, heterogeneity in tissue sampling (e.g., whole muscle vs. specific fiber types), and variability across epigenetic measurement techniques such as bisulfite sequencing, ChIP-based assays, or RNA-seq, which differ in sensitivity and coverage. Population-specific factors—such as age, sex, disease status, training experience, and metabolic profile—further contribute to divergent findings, as do differences in training intensity, volume, and duration used across studies. Collectively, these considerations highlight the need for standardized protocols, larger and more diverse cohorts, and integrative multi-omics approaches to better define the reliability and translational relevance of exercise-induced epigenetic modifications.

## 5 Limitations and research gaps

Current evidence on cardiovascular epigenetic adaptations to RT is limited by substantial heterogeneity in RT protocols across studies, including differences in intensity, volume, duration, and participant characteristics, which complicate comparison and interpretation. Moreover, there is a scarcity of studies assessing epigenetic changes directly in human cardiac or vascular tissues, as most mechanistic insights come from skeletal muscle or preclinical models, restricting the ability to generalize findings to the cardiovascular system. Many investigations also rely on peripheral biomarkers—such as circulating microRNAs or leukocyte DNA methylation—as indirect indicators of cardiovascular adaptations, although these measures may not accurately reflect tissue-specific epigenetic modifications. Additional methodological constraints, including small sample sizes, short intervention periods, variability in analytical techniques, and limited longitudinal designs, further hinder reproducibility and weaken the current evidence base. Therefore, future research should prioritize standardized RT protocols, incorporate tissue-specific assessment approaches, and conduct well-controlled clinical trials to better elucidate how RT modulates epigenetic regulation in cardiovascular tissues.

## 6 Conclusion

Understanding these epigenetic mechanisms opens new perspectives for the prevention and treatment of cardiovascular diseases. Adopting a lifestyle that includes regular RT can be an effective strategy for shaping epigenetic markers favorable to cardiovascular health, highlighting the importance of epigenetic interventions in promoting a healthy heart. Although this review is limited by the many differences in RT protocols in literature, which makes interpreting the results difficult, studies show several beneficial effects of RT on the cardiovascular system with alterations in signaling pathways through epigenetic regulations. Thus, it is essential to continue exploring how RT can be optimized to maximize its epigenetic benefits and improve cardiovascular health in different populations.

However, important limitations of RT must also be acknowledged. RT-induced epigenetic adaptations can vary substantially depending on training intensity, volume, and rest intervals, which are not standardized across studies and may lead to inconsistent cardiovascular outcomes. Additionally, most available evidence relies on skeletal muscle samples, limiting the direct extrapolation of findings to cardiac or vascular tissues. Interindividual variability—driven by age, sex, metabolic status, and pre-existing disease—further complicates the interpretation of RT-specific effects. Moreover, the acute mechanical load imposed by RT may not be suitable for all clinical populations, particularly those with uncontrolled hypertension or structural cardiomyopathies, potentially restricting the generalizability of RT-based epigenetic interventions. These factors highlight the need for more rigorous, mechanistically oriented studies to clarify the specific role of RT in modulating cardiovascular epigenetics. Furthermore, a therapy based on RT-induced epigenetic modification could be implemented in the future to promote cardiovascular health.
